# Prevalence of dyslipidaemia and risk factors in Chinese coal miners: a cross-sectional survey study

**DOI:** 10.1186/s12944-017-0548-9

**Published:** 2017-08-23

**Authors:** Ye Fan, Jian-Jun Huang, Chen-Ming Sun, Nan Qiao, Hai-Xia Zhang, Hui Wang, Ran Tao, Ya-Nan Shen, Tong Wang

**Affiliations:** 1 Department of Environmental Health, School of Public Health, Shanxi Medical University, 56 Xinjiannanlu Street, TaiYuan, Shanxi 030001 China; 2 Department of Surgery, General Hospital of Datong Coal Mining Group, DaTong, Shanxi China; 3 Department of Health Statistics, School of Public Health, Shanxi Medical University, TaiYuan, Shanxi China; 4 Institute for Bacteria disease prevention and control & disinfection, Hebei province center for disease control and prevention, ShiJiazhuang, Hebei China

**Keywords:** Dyslipidaemia, Coal miners, Prevalence, Risk factors

## Abstract

**Background:**

Although coal miners are susceptible to dyslipidaemia owing to their highly risky and stressful working environment as well as unhealthy lifestyle, very few studies have focused on this issue thus far. Therefore, this study investigated the current epidemiological characteristics of dyslipidaemia among Chinese coal miners.

**Methods:**

Demographic, anthropometric, and biochemical data were gathered from 4341 coal miners in China. Dyslipidaemia was diagnosed based on the serum lipid levels. Univariate and multivariate logistic regression analyses were used to assess the related risk factors for dyslipidaemia.

**Results:**

The average concentrations of total cholesterol (TC), triglyceride (TG), high-density lipoprotein cholesterol (HDL-C), and low-density lipoprotein cholesterol (LDL-C) were 5.01 ± 0.93 mmol/L, 1.90 ± 1.72 mmol/L, 1.21 ± 0.35 mmol/L, and 3.15 ± 0.80 mmol/L, respectively. Additionally, 38.08% of participants had a high TC level, 25.84% had a low HDL-C level, 35.08% had a high LDL-C level, and 40.46% had a high TG level. The overall prevalence of dyslipidaemia was 68.28% (95% CI: 66.90–69.66%). Factors associated with dyslipidaemia were age, sex, marital status, monthly family income, type of work, length of service, smoking status, smoking index, drinking status, alcohol consumption per day, elevated fasting glucose, hypertension, obesity and abdominal obesity.

**Conclusions:**

Our study’s results indicated a very high prevalence of dyslipidaemia among Chinese coal miners and identified various risk factors for dyslipidaemia.

## Background

Cardiovascular disease (CVD) accounts for more than half of all non-communicable diseases and has become the leading cause of death worldwide [1]. Large cohort studies dating back to the Framingham Heart Study have identified cholesterol as a modifiable risk factor, which can be treated with lifestyle and pharmacological interventions [2]. Several clinical trials have demonstrated that treatment for dyslipidaemia is effective for preventing CVD [3, 4], and the treatment of dyslipidaemia can reduce the CVD risk by nearly 30% over a 5-year period [5].

Underground coal mining is still one of the most risky occupations globally. Data derived from decennial census and the experience of life insurance companies clearly revealed that death rates for American coal miners are among the highest of any occupational group. When the excess mortality due to respiratory diseases and accidents, which are recognized as hazards related to coal mining, is eliminated, mortality rates for coal miners remain high from 1.4 to 1.7 times the death rates for all workingmen. Excess mortality from other conditions, digestive disorders, certain cancers, and cardiovascular diseases may represent previously unrecognized risks related to coal mining [6]. As non-communicable diseases (NCDs) constitute an increasing portion of the global burden of disease, mining industry employees may be exposed to various factors potentially elevating their NCD risk. Underground coal miners work in a limited space and perform various tasks, and they constantly face a wide range of threats from physical factors, such as a high temperature, humidity, noise, vibration, and radiation [7]. Additionally, all these physical factors were related with NCD. Chronic occupational exposure to noise and vibration were associated with the excess mortality risk for acute myocardial infarction [8, 9]. Dust was also a significant physical factor that workers underground in the mining industry face, as short-term exposure to dust (eg. particulate matter 2.5) increases the risk for hospital admissions for cardiovascular and respiratory diseases [10]. Metabolic risk indicator rates were markedly high and increased significantly from baseline over the 5-year follow-up of a cohort of copper–gold mining company workers, with increased cholesterol, blood pressure, and blood glucose levels and overweight/obesity [11]. A study performed among workers from an open-cast iron ore mine in South Goa suggested an association between the occupation in mining with pneumoconiosis and hearing loss, and it showed that the prevalence rates of diabetes, hypertension, dyslipidaemia and polycythemia were 5.1, 8.3, 37.5, and 12.7%, respectively [12]. Aldehydes such as acrolein are ubiquitous pollutants present in automobile exhaust, cigarette, wood, and coal smoke. Oral exposure to acrolein could induce or exacerbate dyslipidaemia, as could increase levels of plasma cholesterol and triglycerides, thereby contributing to the risk of cardiovascular disease [13].

Owing to the long exposure to coal dust, coal miners are usually affected by disorders of lipid metabolism [14] and susceptible to CVDs [15]; in addition to their working environment, their lifestyle also plays an important role in their susceptibility to CVDs. Findings from open-pit workers indicated a relatively high incidence of hypertension (28%). The level of arterial hypertension is consistently associated with age, length of occupational experience, and body weight, and high prevalence of cardiovascular risk factors: alcohol consumption, cigarette smoking, family history, consumption of salty foods, and overweight among mining and milling workers [16]. The potential risk factors for dyslipidaemia include genetics, age, diet, smoking, physical activity, and stress [17]. Obviously, adopting a healthy lifestyle with proper medication can control these risk factors and their harmfulness to the body. Therefore, an important public health priority is to prevent dyslipidaemia and its related risk factors. Researchers have shown that the risk of CVD increases as the exposure to coal mine dust and other coal mine risk factors increases [15, 18], and an underground work environment in a coal mine is a risk factor for lipid metabolic abnormalities [19]. Therefore, knowledge of the current magnitude of dyslipidaemia in Chinese coal miners is important for enhanced occupational protection measures and prevention and management of CVD, in turn to effectively reduce occupational hazards. However, surveys to determine the burden of dyslipidaemia among Chinese coal miners have not been conducted in the last few years, and available data on the prevalence, types, and related factors of dyslipidaemia in this occupational population are rather limited and outdated. We hypothesized that remarkable changes in the prevalence, type, and risk factors of dyslipidaemia among Chinese coal miners would happen in recent years. Therefore, the purpose of this study was to obtain present data on dyslipidaemia, including its prevalence and risk factors, in coal miners in China.

## Methods

### Study population

Study participants were recruited from the Datong Coal Mine Group, with an estimated 200,000 permanent staff members at 87 coal mines. We obtained the baseline data of all staff from the administrative department of the coal mine group, which included the name, sex, date of birth, and work type to build the sampling frame. Based on these data, study subjects were selected using a two-stage cluster sampling. In the first phase, we randomly sampled 10 coal mines from 87 coal mines as the primary sampling unit. The number of permanent staff members in these sampled coal mines was approximately 38,951. In the second stage, stratified random sampling was applied according to the factors, including the work place (underground front-line, underground auxiliary and surface), age (20–65 years, with groups of 5-year intervals), and sex (male or female). SAS software, version 9.2 (SAS Institute, Cary, NC, USA) was used to perform Surveyselect procedure. The proportion of the sampling population was 11.2%. Considering no response or other non-compliance issues, we administered a questionnaire to 4400 miners, and 4341 of them returned completed questionnaires from August 1, 2013 to December 30, 2013.

### Physical examinations

The physical examination was performed by trained physicians at the General Hospital of the Datong Coal Mining Group. With the subjects wearing light weight clothes and no shoes, their weight and height were measured using a height-measuring and weight-measuring instrument. We calculated the body mass index (BMI) by dividing weight in kilograms by height in meters squared. With the subjects standing and breathing normally, their waist circumference (WC) was measured at the umbilicus, and hip circumference was measured around the buttocks. The waist-hip ratio (WHR) was calculated using the following formula: waist circumference (cm)/hip circumference (cm).

### Blood sample collection and biochemistry analysis

For all participants, fasting blood samples were collected in the morning after fasting at least 8 h. The lipid panel, including total cholesterol (TC), triglyceride (TG), high-density lipoprotein cholesterol (HDL-C), and low-density lipoprotein cholesterol (LDL-C), serum fasting blood glucose (FBG), along with other routine blood biochemical indexes were determined by routine enzymatic methods on automated modular analysers (Siemens Advia 2400 Chemistry Analyser, Diamond Diagnostic, Holliston, MA, USA) at the General Hospital of the Datong Coal Mining Group.

### Definitions of dyslipidaemia, obesity, FBG, and hypertension

TC, LDL-C, HDL-C, and TG levels were classified based on the 2016 Chinese guideline for the management of dyslipidaemia in adults [20]. Borderline high and high TC was defined as TC levels of 5.2–6.2 mmol/L (200–240 mg/dL) and ≥6.2 mmol/L (≥240 mg/dL), respectively, and a TC level < 5.2 mmol/L (<200 mg/dL) was defined as acceptable. Low HDL-C was defined as HDL-C levels <1.0 mmol/L (<40 mg/dL). Borderline high and high LDL-C was defined as LDL-C levels of 3.4–4.1 mmol/L (130–160 mg/dL) and ≥4.1 mmol/L (≥160 mg/dL), respectively, and LDL-C levels <3.4 mmol/L (130 mg/dL) and <2.6 mmol/L (100 mg/dL) were defined as acceptable and ideal, respectively. Borderline high and high TG was defined as TG levels of 1.7–2.3 mmol/L (150–200 mg/dL) and ≥2.3 mmol/L (≥200 mg/dL), respectively, and a TG level < 1.7 mmol/L (150 mg/dL) was defined as acceptable.

The Ministry of Health of China defines the following four categories of body weight based on BMI values: underweight, <18.5 kg/m^2^; normal weight, 18.5–23.9 kg/m^2^; overweight, 24.0–27.9 kg/m^2^; and obesity, ≥28.0 kg/m^2^ [21]. When the WCs are ≥88 cm for women and ≥102 cm for men, individuals are considered to have “abdominal obesity”. In addition, hypso-WHR is defined when the WHRs are >0.9 for men and >0.8 for women [22].

According to the American Diabetes Association, study participants were categorised into three groups based on FBG levels [23]: normal fasting glucose (NFG; FBG < 5.6 mmol/L (100 mg/dL)), impaired fasting glucose (IFG; 5.6 mmol/L (100 mg/dL) ≤ FBG < 7.0 mmol/L (126 mg/dL)), and diabetes mellitus (DM; FBG ≥ 7.0 mmol/L (126 mg/dL)).

After avoiding exercise and caffeinated beverages for at least one-half hour before measuring blood pressure, the systolic blood pressure (SBP) and diastolic blood pressure (DBP) of the participants were obtained using a mercury sphygmomanometer (GB3053–93 Yuyue, Armamentarium Limited Company, Jiangsu, China). A high blood pressure was defined as a SBP ≥ 140 mmHg and/or DBP ≥ 90 mmHg [24].

### Questionnaire

A survey was conducted by trained investigators after the physical examination. Data were collected by gathering all participants in a conference room along with cardiologists and trained nurses. All participants agreed to participate in a face-to-face interview using a standard questionnaire. Participants’ social demographic characteristics (sex, age, etc.), work situation (workplace, work type, etc.), lifestyle and behaviours, medical history, smoking and drinking status, dietary habits, monthly family income, marital status, and educational level were determined by the structured questionnaire.

### Statistical analyses

Data entry was conducted using EpiData 3.0 software (EpiData Association, Odense, Denmark), and to reduce error in generating an electronic data set, we used double data entry. Descriptive analyses of all variables were performed (mean ± standard deviation for continuous data and frequencies or percentages for categorical variables). Comparisons of continuous variables were made using the unpaired Student’s t test. The Pearson’s chi-square test was used to evaluate differences in the categorical variables. Furthermore, the prevalence of an abnormal lipid panel according to various characteristics was calculated. Univariate and multivariate logistic regression analyses adjusted for age and sex were used to evaluate the association between blood lipid levels and risk factors, with abnormal TC, TG, HDL-C, and LDL-C levels as dependent variables and demographic characteristics, working characteristics, lifestyles and behaviours, anthropometric characteristics, and biochemical indices as independent variables (entry = 0.05, removal = 0.10). The association between dyslipidaemia and risk factors was assessed by odds ratios (ORs), and the adjusted ORs were presented with 95% confidence intervals. To further assess major risk factors for the prevalence of dyslipidaemia and control for potential confounders, we performed stepwise multiple logistic regression analysis to identify the risk factors for each type of dyslipidaemia, adjusted for age and sex. SAS software, version 9.4 (SAS Institute, Cary, NC, USA) was used to perform all statistical analyses. There was a significance difference when *P* < 0.05, and all *P*-values were two-tailed.

### Ethical considerations

This study was approved by the ethics committee of Shanxi Medical University (ethical application reference: HX201201). All study processes were conducted according to the ethical criteria. After all participants were informed of the study’s purpose, medical programs, benefits, and non-disclosure agreement of individual information, they signed a written consent.

## Results

### Characteristics of the study population

Overall, 4341 coal miners from 10 coal mines were sampled. The survey population accounted for 2.2% of the total participants. The mean (standard deviation) age of the subjects was 41.79 (8.64) years, and men accounted for 84.22% (3656/4341) of the population. There were 908 (21.01%) underground front-line participants, 1497 (34.64%) underground auxiliary participants, 1118 (25.87%) ground participants and 798 (18.47%) office participants. The mean levels of TC, TG, HDL-C, and LDL-C were 5.01 ± 0.93 mmol/L, 1.90 ± 1.72 mmol/L, 1.21 ± 0.35 mmol/L, and 3.15 ± 0.80 mmol/L, respectively. Demographic, clinical, and work characteristics of participants enrolled in this cross-sectional occupational population-based survey according to their dyslipidaemia status are shown in Table 1. The percentages of men, married participants, drinkers and smokers were all significantly higher in the dyslipidaemia group than in the control group, whereas the percentages of intense physical workers and underground front-line workers were lower (*P* < 0.001, except *P* < 0.05 for marital status and workplace). The dyslipidaemia group was older; had a higher FBG, SBP, DBP, BMI, waist circumferences, WHR, TC level, TG level, and LDL-C level; and had a lower HDL-C level compared to the non-dyslipidaemia group (all *P* < 0.001).

**Table 1:** Characteristics of participants according to dyslipidemia status

Characteristics	All subjects	With dyslipidemia					Without dyslipidemia	t/χ^2^	*P value*
		Overall	High TG (≥1.7 mmol/L)	High TC (≥5.2 mmol/L)	High LDL-C(≥3.4 mmol/L)	Low HDL-C (<1.0 mmol/L)			
All, n (%)	4341(100)	2964(68.28)	1746(40.46)	1643(38.08)	1523(35.08)	1115(25.84)	1377(31.72)		
Age (years), mean (SD)	41.79(8.64)	42.42(8.57)	42.46(8.34)	43.09(8.36)	43.20(8.49)	41.22(8.91)	40.43(8.64)	−7.11	<0.001
Male, n (%)	3656(84.22)	2609(71.36)	1584(43.46)	1411(38.71)	1334(36.49)	1045(28.67)	1047(28.64)	102.27	<0.001
Married, n (%)	4057(93.80)	2792(68.82)	1664(41.28)	1551(38.48)	1440(35.49)	1034(25.65)	1265(31.18)	9.65	0.002
Education, n (%)									
Primary school or under	116(2.69)	80(68.97)	48(41.38)	47(40.52)	40(34.48)	29(25.00)	36(31.03)	0.41	0.939
Junior high school	1092(25.29)	738(67.58)	428(39.74)	435(40.39)	390(35.71)	240(22.28)	354(32.42)		
Junior college or Senior high school	2601(60.24)	1784(68.59)	1059(40.87)	967(37.32)	917(35.26)	687(26.51)	817(31.41)		
Bachelor degree or above	509(11.79)	346(67.98)	205(40.35)	182(35.83)	166(32.61)	156(30.71)	163(32.02)		
Monthly income (RMB, Yuan), n (%)									
≤ 4000	1127(26.28)	774(68.68)	436(39.00)	438(39.18)	399(35.40)	305(27.28)	353(31.32)	1.39	0.499
4000–6000	1803(42.05)	1240(68.77)	752(41.94)	722(40.27)	662(36.72)	408(22.76)	563(31.23)		
>6000	1358(31.67)	909(66.94)	537(39.75)	455(33.68)	439(32.33)	395(29.24)	449(33.06)		
Working type, n (%)									
Intense physical workers	1124(26.28)	708(62.99)	390(34.98)	404(36.23)	355(31.58)	238(21.35)	416(37.01)	22.61	<0.001
Light physical workers	2095(48.98)	1491(71.17)	906(43.56)	803(38.61)	753(35.94)	596(28.65)	604(28.83)		
Mental workers	1058(24.74)	721(68.15)	428(40.53)	404(38.26)	389(36.77)	268(25.38)	337(31.85)		
Workplace, n (%)									
Underground front-line	908(21.01)	580(63.88)	321(35.55)	311(34.44)	288(31.72)	209(23.15)	328(36.12)	16.13	0.001
Underground auxiliary	1497(34.64)	1063(71.01)	671(45.00)	568(38.10)	519(34.67)	414(27.77)	434(28.99)		
Ground	1118(25.87)	785(70.21)	437(39.09)	464(41.50)	425(38.01)	304(27.19)	333(29.79)		
Office	798(18.47)	532(66.67)	314(39.35)	297(37.22)	290(36.34)	188(23.56)	266(33.33)		
Habitual drinking, n(%)	1795(41.49)	1329(74.04)	838(46.84)	762(42.59)	660(36.77)	442(24.71)	466(25.96)	46.91	<0.001
Current smoking, n(%)	2495(57.69)	1815(72.75)	1090(43.79)	1006(40.42)	948(38.00)	722(29.01)	680(27.25)	54.35	<0.001
FBG (mmol/L), mean (SD)	5.00(1.30)	5.10(1.47)	5.16(1.57)	5.22(1.53)	5.23(1.55)	5.09(1.61)	4.78(0.78)	−9.28	<0.001
SBP(mmHg), mean (SD)	126.95(17.62)	129.0(17.40)	130.3(17.56)	129.7(17.74)	129.8(17.68)	128.2(16.61)	122.6 (17.31)	−11.27	<0.001
DBP(mmHg), mean (SD)	78.85(14.18)	80.56(14.18)	81.93(14.37)	80.67(14.42)	81.04(14.27)	80.72(14.02)	75.17(13.46)	−12.01	<0.001
BMI (kg/m^2^), mean (SD)	24.92(5.97)	25.56(3.63)	26.27(3.67)	25.37(3.50)	25.81(3.44)	26.41(3.78)	23.52(9.01)	−8.05	<0.001
WC (cm), mean (SD)	89.97(9.81)	91.76(9.51)	93.30(9.17)	91.40(9.61)	92.06(9.47)	93.71(8.91)	86.09(9.34)	−18.16	<0.001
WHR, mean (SD)	0.89(0.08)	0.90(0.07)	0.91(0.07)	0.90(0.07)	0.90(0.06)	0.91(0.07)	0.88(0.09)	−8.4	<0.001
TC (mmol/L), mean (SD)	5.01(0.93)	5.28(0.96)	5.29(0.98)	5.94(0.67)	5.85(0.74)	4.73(0.94)	4.41(0.49)	−39.85	<0.001
TG (mmol/L), mean (SD)	1.90(1.72)	2.31(1.92)	3.09(2.18)	2.41(2.36)	2.15(1.61)	2.71(2.37)	1.00(0.31)	−36.1	<0.001
HDL-C (mmol/L), mean (SD)	1.21(0.35)	1.15(0.36)	1.07(0.30)	1.30(0.38)	1.21(0.31)	0.83(0.14)	1.36(0.26)	21.34	<0.001
LDL-C (mmol/L), mean (SD)	3.15(0.80)	3.41(0.77)	3.37(0.79)	3.84(0.64)	3.99(0.51)	3.10(0.71)	2.57(0.47)	−43.51	<0.001

*P-value for comparison between subjects with and without dyslipidemia at α = 0.05 level*

*FBG* fasting blood glucose, *SBP* systolic blood pressure, *DBP* diastolic blood pressure, *BMI* body mass index, *WC* waist circumferenc

*WHR* waist/hip ratio, *H-TC* high total cholesterol, *H-TG* high triglyceride, *H-LDL* high low-density lipoprotein cholesterol, *L-HDL* low high-density lipoprotein cholesterol

### Prevalence of dyslipidaemia

As shown in Tables 1 and 2, 68.28% (95% CI: 66.90–69.66%) of Chinese coal miners had at least one type of dyslipidaemia (including borderline high and high TC, LDL-C, and TG levels and a low HDL-C level; men, 71.36%; women, 51.68%). Analysis of the dyslipidaemia components showed that 38.08% of the population had a high TC level, 40.46% had a high TG level, 35.08% had an increased LDL-C level, and 25.84% had an abnormally low HDL-C level. The prevalence rates of borderline high and high TC levels were 27.69 and 10.38%, respectively; those of borderline high and high LDL-C levels were 23.15 and 11.93%, respectively; that of a low HDL-C level was 25.84%; and those of borderline high and high TG levels were 16.22 and 24.24%, respectively. The age-specific prevalence of borderline high and high TC, LDL-C, and TG levels increased with increasing age (*P* < 0.001). The prevalences of borderline high and high TC, LDL-C, and TG levels and a low HDL-C level were significantly higher in men than in women, but they were significantly lower in the underground than in the surface workplace and lower in the intense physical work type (*P* < 0.05).

**Table 2:** Proportion of concentrations classification of TC, TG, HDL-C and LDL-C in Chinese coal miners (mmol/L)

Variable (Characteristics)	TC			LDL-C				HDL-C		TG		
	<5.2	5.2–6.2	≥6.2	<2.6	2.6–3.4	3.4–4.1	≥4.1	<1.0	≥1.0	<1.7	1.7–2.3	≥2.3
All, n(%)	2672(61.92)	1195(27.69)	448(10.38)	1143(26.33)	1675(38.59)	1005(23.15)	518(11.93)	1115(25.84)	3200(74.16)	2569(59.54)	700(16.22)	1046(24.24)
Age(years), n(%)												
< 40	1254(66.88)	471(25.12)	150(8.00)	559(29.72)	754(40.09)	370(19.67)	198(10.53)	509(27.15)	1366(72.85)	1170(62.40)	285(15.20)	420(22.40)
≥ 40	1418(58.11)	724(29.67)	298(12.21)	584(23.74)	921(37.44)	635(25.81)	320(13.01)	606(24.84)	1834(75.16)	1399(57.34)	415(17.01)	626(25.66)
*P value*	<0.001			<0.001				0.086		0.001		
Gender, n(%)												
Male	2234(61.29)	1024(28.09)	387(10.62)	913(24.97)	1409(38.54)	876(23.96)	458(12.53)	1045(28.67)	2600(71.33)	2061(56.54)	617(16.93)	967(26.53)
Female	438(65.47)	171(25.56)	60(8.97)	230(33.63)	266(38.89)	128(18.71)	60(8.77)	70(10.46)	599(89.54)	508(75.93)	83(12.41)	78(11.66)
*P value*	0.04			<0.001				<0.001		<0.001		
Working type, n(%)												
Intense physical workers	711(63.77)	305(27.35)	99(8.88)	323(28.74)	446(39.68)	242(21.53)	113(10.05)	238(21.35)	877(78.65)	725(65.02)	164(14.71)	226(20.27)
Light physical workers	1277(61.39)	577(27.74)	226(10.87)	539(25.73)	803(38.33)	499(23.82)	254(12.12)	596(28.65)	1484(71.35)	1174(56.44)	357(17.16)	549(26.39)
Mental workers	652(61.74)	292(27.65)	112(10.61)	267(25.24)	402(38.00)	247(23.35)	142(13.42)	268(25.38)	788(74.62)	628(59.47)	175(16.57)	253(23.96)
*P value*	0.205			0.007				<0.001		<0.001		
Workpalce, n(%)												
Underground	592(65.56)	232(25.69)	79(8.75)	266(29.30)	354(38.99)	200(22.03)	88(9.69)	209(23.15)	694(76.85)	582(64.45)	122(13.51)	199(22.04)
Front-line												
Underground auxiliary	923(61.90)	416(27.90)	152(10.19)	360(24.05)	618(41.28)	341(22.78)	178(11.89)	414(27.77)	1077(72.23)	820(55.00)	272(18.24)	399(26.76)
Ground	654(58.50)	333(29.79)	131(11.72)	293(26.21)	400(35.78)	278(24.87)	147(13.15)	304(27.19)	814(72.81)	681(60.91)	174(15.56)	263(23.52)
Office	501(62.78)	211(26.44)	86(10.78)	208(26.07)	300(37.59)	185(23.18)	105(13.16)	188(23.56)	610(76.44)	484(60.65)	131(16.42)	183(22.93)
*P value*	0.011			0.015				0.023		<0.001		

Data are n (%). *TC* total cholesterol, *HDL-C* high-density lipoprotein cholesterol, *LDL-C* low-density lipoprotein cholesterol, *TG* triglyceride

### Factors associated with dyslipidaemia

The prevalence rate of abnormal serum lipid concentrations in accordance with participants’ different demographic, work, lifestyle, and behaviour characteristics and the results of univariate logistic analyses of factors associated with dyslipidaemia adjusted by age and sex are presented in Tables 3 and 4, respectively. Notably, the high TC, LDL-C, and TG prevalence significantly increased with advanced age (≥40 years), but the low HDL-C prevalence decreased (adjusted ORs = 1.45, 1.45, 1.19, and 0.85, respectively; *P* < 0.05). The ORs for a high LDL-C level, high TG level, and low HDL-C level were lower among women(adjusted ORs = 0.68, 0.42, and 0.29, respectively; *P* < 0.001). Only the high prevalence of high TG levels was related to the marital status: married participants had a higher OR of high TG levels than single participants (adjusted OR = 1.63; *P* < 0.001). Subjects with a high family income were more likely to have lower ORs of high TC level (>6000 yuan: adjusted OR = 0.79; *P* < 0.05) and low HDL-C level (4000–6000 yuan: adjusted OR = 0.66; *P* < 0.001).

**Table 3:** Prevalence of dyslipidemia by demographic and working characteristics and adjusted odds ratios (95%CI) of dyslipidemia prevalence

Variable	Characteristics	High TC(≥5.2 mmol/L)		High LDL-C(≥3.4 mmol/L)		Low HDL-C (<1.0 mmol/L)		High TG(≥1.7 mmol/L)	
		Adjusted	*P*	Adjusted	*P*	Adjusted	*P*	Adjusted	*P*
		OR (95% CI)		OR (95% CI)		OR (95% CI)		OR (95% CI)	
Age(years)	<40	1		1		1		1	
	≥40	1.45(1.27, 1.64)	<0.001	1.45(1.27, 1.64)	<0.001	0.85(0.74, 0.97)	0.018	1.19(1.05, 1.35)	0.005
Gender	Male	1		1		1		1	
	Female	0.86(0.72, 1.02)	0.09	0.68 (0.56, 0.81)	<0.001	0.29(0.22, 0.37)	<0.001	0.42(0.35, 0.51)	<0.001
Marital status	Single	1		1		1		1	
	Married	1.22(0.93, 1.60)	0.152	1.18(0.90, 1.56)	0.238	0.87(0.66, 1.16)	0.351	1.63(1.23, 2.15)	<0.001
Education	Primary school or under	1		1		1		1	
	Junior high school	1.05(0.71, 1.55)	0.805	1.13(0.75, 1.69)	0.564	0.87(0.56, 1.36)	0.536	0.98(0.66, 1.45)	0.918
	Junior college or Senior high school	1.03(0.70, 1.51)	0.896	1.25(0.84, 1.85)	0.273	1.12(0.72, 1.74)	0.608	1.13(0.77, 1.66)	0.529
	Bachelor degree or above	1.04(0.68, 1.59)	0.851	1.23(0.79, 1.89)	0.357	1.48 (0.92, 2.37)	0.108	1.23(0.80, 1.87)	0.345
Monthly income	≤4000	1		1		1		1	
(RMB, Yuan)	4000–6000	1.02(0.87, 1.19)	0.793	0.99(0.85, 1.17)	0.96	0.66(0.55, 0.79)	<0.001	0.99(0.85, 1.16)	0.956
	>6000	0.79(0.67, 0.94)	0.006	0.86(0.73, 1.02)	0.084	1.01(0.84, 1.21)	0.946	0.98(0.83, 1.16)	0.814
Working type	Intense physical workers	1		1		1		1	
	Light physical workers	1.14(0.98, 1.32)	0.102	1.29(1.10, 1.51)	0.001	1.68(1.41, 1.99)	<0.001	1.60(1.38, 1.87)	<0.001
	Mental workers	1.18(0.98, 1.42)	0.079	1.48(1.23, 1.79)	<0.001	1.73(1.41, 2.13)	<0.001	1.70(1.41, 2.04)	<0.001
Workplace	Underground front-line	1		1		1		1	
	Underground auxiliary	1.17(0.98, 1.39)	0.08	1.14(0.96, 1.36)	0.147	1.29(1.06, 1.56)	0.01	1.49(1.25, 1.76)	<0.001
	Ground	1.41(1.17, 1.71)	<0.001	1.49(1.23, 1.81)	<0.001	1.76(1.43, 2.18)	<0.001	1.50(1.24, 1.81)	<0.001
	Office	1.22(0.98, 1.51)	0.073	1.49(1.20, 1.85)	<0.001	1.68(1.33, 2.14)	<0.001	1.73(1.40, 2.14)	<0.001
Length of service	≤3 years	1		1		1		1	
	4–10 years	1.26(0.82, 1.95)	0.29	1.39(0.89, 2.18)	0.152	1.14(0.75, 1.72)	0.552	0.99(0.66, 1.48)	0.959
	>10 years	1.87(1.25, 2.79)	0.002	1.98(1.30, 3.02)	0.001	0.87(0.59, 1.29)	0.496	1.39(0.96, 2.01)	0.083

*TC* total cholesterol, *HDL-C* high-density lipoprotein cholesterol, *LDL-C* low-density lipoprotein cholesterol, *TG* triglyceride

**Table 4:** Prevalence of dyslipidemia by lifestyle and behavior characteristics and adjusted odds ratios (95%CI) of dyslipidemia prevalence

Variable	Characteristics	High TC (≥5.2 mmol/L)		High LDL-C (≥3.4 mmol/L)		Low HDL-C (<1.0 mmol/L)		High TG (≥1.7 mmol/L)	
		Adjusted	*P*	Adjusted	*P*	Adjusted	*P*	Adjusted	*P*
		OR (95% CI)		OR (95% CI)		OR (95% CI)		OR (95% CI)	
Smoking status	No	1		1		1		1	
	Yes	1.28(1.10, 1.48)	0.001	1.24(1.07, 1.44)	0.004	1.06(0.91, 1.24)	0.48	1.05(0.91, 1.21)	0.472
Smoking index (roots*years)	0~	1		1		1		1	
	150~	1.41(1.17, 1.70)	<0.001	1.34(1.11, 1.61)	0.002	0.98(0.80, 1.20)	0.847	1.07(0.89, 1.29)	0.454
	300~	1.18(0.95, 1.47)	0.13	1.22(0.98, 1.52)	0.076	1.00(0.79, 1.27)	0.979	1.07(0.86, 1.32)	0.562
	450~	1.35(1.12, 1.62)	0.001	1.37(1.14, 1.65)	<0.001	1.21(0.99, 1.48)	0.064	1.15(0.96, 1.38)	0.142
Passive smoking	No	1		1		1		1	
	Yes	1.09(0.95, 1.25)	0.228	1.11(0.96, 1.27)	0.152	1.11(0.95, 1.30)	0.189	1.11(0.97, 1.28)	0.133
Alcohol Drinking Status	No	1		1		1		1	
	Yes	1.39(1.21, 1.58)	<0.001	1.04(0.91, 1.18)	0.612	0.70(0.61, 0.81)	<0.001	1.33(1.17, 1.52)	<0.001
Daily drinking(g)	0~	1		1		1		1	
	25~	1.52(1.19, 1.96)	0.001	1.08(0.83, 1.39)	0.572	0.69(0.52, 0.93)	0.013	1.10(0.85, 1.41)	0.479
	50~	1.99(1.46, 2.71)	<0.001	1.29(0.95, 1.75)	0.107	0.38(0.25, 0.58)	<0.001	0.99(0.72, 1.34)	0.923
	75~	1.89(1.45, 2.45)	<0.001	0.90(0.69, 1.18)	0.449	0.49(0.35, 0.68)	<0.001	1.67(1.28, 2.17)	<0.001
Sleep time	Sufficient(> = 8 h)	1		1		1		1	
	Insufficient(<8 h)	0.98(0.86, 1.11)	0.745	0.96(0.85, 1.09)	0.554	0.89(0.78, 1.03)	0.111	0.95(0.83, 1.07)	0.38
Eat speed	Fast	1		1		1		1	
	Moderate	1.05(0.92, 1.20)	0.441	0.92(0.80, 1.05)	0.208	0.78(0.68, 0.91)	0.001	0.88(0.77, 1.00)	0.052
	Slow	1.07(0.86, 1.34)	0.554	0.83(0.66, 1.04)	0.105	0.55(0.42, 0.72)	<0.001	0.72(0.58, 0.90)	0.005

*TC* total cholesterol, *HDL-C* high-density lipoprotein cholesterol, *LDL-C* low-density lipoprotein cholesterol, *TG* triglyceride

Participants who did light physical and mental work were more likely to have higher ORs of high LDL-C levels (adjusted ORs = 1.29, 1.48, respectively; *P* < 0.05), high TG levels (adjusted ORs = 1.60, 1.70, respectively; *P* < 0.001) and low HDL-C levels (adjusted ORs = 1.68, 1.73, respectively; *P* < 0.001) than those who did intense physical labour. Further, compared to the underground front-line work group, the surface work place group were more inclined to suffer from high TC (ground: adjusted OR =1.41; *P* < 0.001), high LDL-C (ground: adjusted OR =1.49; *P* < 0.001; office: adjusted OR =1.49; *P* < 0.001), high TG (adjusted ORs for underground auxiliary, ground and office =1.49, 1.50, 1.73, respectively; *P* < 0.001) and low HDL-C (adjusted ORs for underground auxiliary, ground and office =1.29, 1.76, 1.68, respectively; *P* < 0.05). Additionally, having an extended length of service (>10 years of service) increased the tendency of high TC (adjusted OR =1.87; *P* < 0.05) and high LDL-C levels (adjusted OR =1.98; *P* < 0.05).

Cigarette smoking and the prevalence of high TC and high LDL-C levels showed a positively relationship (adjusted ORs = 1.28 and 1.24, respectively; *P* < 0.05). In addition, participants with a bigger smoking index had a greater prevalence of high TC (150 ~ and 450~: adjusted ORs = 1.41 and 1.35, respectively; *P* < 0.05) and high LDL-C levels (150 ~ and 450~: adjusted ORs = 1.34 and 1.37, respectively; *P* < 0.05). The prevalence of high TC and high TG levels was higher among current drinkers than non-drinkers, but the trend was opposite for low HDL-C levels (adjusted ORs = 1.39, 1.33, and 0.70, respectively; *P* < 0.001). The daily alcohol consumption was positively associated with a high prevalence rate of elevated TC levels (25 ~ g/d, 50 ~ g/d and 75 ~ g/d: adjusted ORs =1.52, 1.99, and 1.89, respectively; *P* < 0.05) and elevated TG levels (75 ~ g/d: adjusted OR = 1.67; *P* < 0.001), but it was inversely related to the prevalence of low HDL-C levels (25 ~ g/d, 50 ~ g/d, and 75 ~ g/d: adjusted ORs = 0.69, 0.38 and 0.49, respectively; *P* < 0.05). Participants with a slower eating speed had lower ORs of low HDL-C levels (moderate and slow eating speed: adjusted ORs = 0.78 and 0.55, respectively; *P* < 0.05) and elevated TG levels (slow eating speed: adjusted OR = 0.72; *P* < 0.05) than participants who had a faster eating speed. Furthermore, there was no significant association of the prevalence of any kind of dyslipidaemia with education level, passive smoking, and sleep time.

Results of univariate logistic analyses of the anthropometric and biochemistry indices associated with dyslipidaemia, adjusted by age and sex, are shown in Table 5. Participants who had elevated fasting glucose levels were more likely to have a higher prevalence rate of high TC levels (IFG: adjusted OR = 1.55, DM: adjusted OR = 2.32; *P* < 0.001), high LDL-C levels (IFG: adjusted OR = 1.40, DM: adjusted OR = 2.06; *P* < 0.05), high TG levels (IFG: adjusted OR = 1.80, DM: adjusted OR = 3.28; *P* < 0.001) and low HDL-C levels (DM: adjusted OR = 1.96; *P* < 0.001). Subjects with hypertension had an increased tendency of high TC levels (SBP: adjusted OR = 1.38, DBP: adjusted OR = 1.45; *P* < 0.001), high LDL-C levels (SBP: adjusted OR = 1.36, DBP: adjusted OR = 1.47; *P* < 0.001), high TG levels (SBP: adjusted OR = 1.65, DBP: adjusted OR = 1.99; *P* < 0.001), and low HDL-C levels (DBP: adjusted OR = 1.24; *P* < 0.05). Additionally, participants with a large body size had a greater prevalence of high TC levels (overweight: adjusted OR = 1.50, obese: adjusted OR = 1.73; *P* < 0.001), high LDL-C levels (overweight: adjusted OR = 1.86, obese: adjusted OR = 2.56; *P* < 0.001), low HDL-C levels (overweight: adjusted OR = 2.09, obese: adjusted OR = 3.77; *P* < 0.001), and high TG levels (overweight: adjusted OR = 2.92, obese: adjusted OR = 5.16; *P* < 0.001). Participants with central obesity tended to suffer high TC levels (large waist: adjusted OR = 1.30, hypso-WHR: adjusted OR = 1.28; *P* < 0.05), high LDL-C levels (large waist: adjusted OR = 1.54, hypso-WHR: adjusted OR = 1.41; *P* < 0.001), low HDL-C levels (large waist: adjusted OR = 2.48, hypso-WHR: adjusted OR = 1.74; *P* < 0.001), and high TG levels (large waist: adjusted OR = 2.86, hypso-WHR: adjusted OR = 1.95; *P* < 0.001).

**Table 5:** Prevalence of dyslipidemia by anthropometric and biochemical parameters and adjusted odds ratios (95%CI) of dyslipidemia prevalence

Variable	Characteristics	High TC (≥5.2 mmol/L)		High LDL-C (≥3.4 mmol/L)		Low HDL-C (<1.0 mmol/L)		High TG (≥1.7 mmol/L)	
		Adjusted OR	*P*	Adjusted OR	*P*	Adjusted OR	*P*	Adjusted OR	*P*
		(95% CI)		(95% CI)		(95% CI)		(95% CI)	
FBG	NFG(<5.6 mmol/L)	1		1		1		1	
	IFG(5.6 mmol/L ≤ FBG < 7.0 mmol/L)	1.55(1.28, 1.89)	<0.001	1.40(1.15, 1.71)	0.001	1.08(0.86, 1.34)	0.515	1.80(1.48, 2.19)	<0.001
	DM(≥7.0 mmol/L)	2.32(1.70, 3.17)	<0.001	2.06(1.51, 2.80)	<0.001	1.96(1.42, 2.70)	<0.001	3.28(2.35, 4.57)	<0.001
SBP	<140 mmHg	1		1		1		1	
	≥140 mmHg	1.38(1.19, 1.61)	<0.001	1.36(1.16, 1.58)	<0.001	1.11(0.93, 1.31)	0.242	1.65(1.42, 1.93)	<0.001
DBP	<90 mmHg	1		1		1		1	
	≥90 mmHg	1.45(1.25, 1.69)	<0.001	1.47(1.26, 1.70)	<0.001	1.24(1.05, 1.46)	0.01	1.99(1.71, 2.31)	<0.001
BMI	normal weight, 18.5–23.9 kg/m^2^	1		1		1		1	
	underweight, <18.5 kg/m^2^	0.99(0.67, 1.46)	0.946	0.65(0.41, 1.03)	0.067	0.56(0.30, 1.03)	0.064	1.10(0.72, 1.68)	0.665
	overweight, 24.0–27.9 kg/m^2^	1.50(1.30, 1.72)	<0.001	1.86(1.61, 2.16)	<0.001	2.09(1.77, 2.48)	<0.001	2.92(2.51, 3.39)	<0.001
	obesity, ≥28.0 kg/m^2^	1.73(1.45, 2.07)	<0.001	2.56(2.14, 3.07)	<0.001	3.77(3.09, 4.59)	<0.001	5.16(4.28, 6.22)	<0.001
WC	<102 cm (male)/88 cm (female)	1		1		1		1	
	≥102 cm (male)/88 cm (female)	1.30(1.08, 1.56)	0.005	1.54(1.28, 1.86)	<0.001	2.48(2.03, 3.03)	<0.001	2.86(2.36, 3.46)	<0.001
WHR	≤0.9(male)/0.8 (female)	1		1		1		1	
	>0.9(male)/0.8 (female)	1.28(1.13, 1.46)	<0.001	1.41(1.23, 1.60)	<0.001	1.74(1.50, 2.00)	<0.001	1.95(1.71, 2.22)	<0.001

*FBG* fasting blood glucose, *NFG* normal fasting glucose, *IFG* impaired fasting glucose, *DM* diabetes mellitus, *TG*, triglyceride, *TC*, total Cholesterol, *HDL-C* high-density lipoprotein cholesterol, *LDL-C* low density lipoprotein cholesterol, *SBP* systolic blood pressure, *DBP* diastolic blood pressure, *BMI* body mass index, *WC* waist circumference, *WHR* waist/hip ratio

### Results of multivariable logistic regression analysis of dyslipidaemia

Data in Table 6 suggest that the presence of high TC levels was still positively associated with an extended length of service (>10 years: adjusted OR = 1.70; *P* < 0.05), smoking status (adjusted OR = 1.25; *P* < 0.05), increased alcohol consumption (25 ~ g/d, 50 ~ g/d and 75 ~ g/d: adjusted ORs = 1.53, 1.81 and 1.73, respectively; *P* < 0.05), elevated fasting glucose (IFG: adjusted OR = 1.41; *P* < 0.05; DM: adjusted OR = 1.99; *P* < 0.001), hypertension (DBP: adjusted OR = 1.23; *P* < 0.05), and bigger body size (overweight: adjusted OR = 1.43, obese: adjusted OR = 1.66; *P* < 0.001), but it was inversely related to the monthly family income (>6000 yuan: adjusted OR = 0.76; *P* < 0.05). Participants who engaged in mental labour (adjusted OR = 1.35; *P* < 0.05), had an extended length of service (>10 years: adjusted OR = 1.77; *P* < 0.05), higher smoking index (150~, 300 ~ and 450~: adjusted ORs = 1.39, 1.28, and 1.42, respectively; *P* < 0.05), elevated fasting glucose (DM: adjusted OR = 1.61; *P* < 0.05), hypertension (DBP: adjusted OR = 1.30; *P* < 0.05), or bigger body size (overweight: adjusted OR = 1.77, obese: adjusted OR = 2.35; *P* < 0.001) had an increased tendency of a high LDL-C level. Table 6 also illustrates that participants who were married (adjusted OR = 1.43; *P* < 0.05), engaged in light physical or mental labour (adjusted ORs = 1.51 and 1.58, respectively; *P* < 0.001), drinking (adjusted OR =1.24; *P* < 0.05), had increased alcohol consumption (75 ~ g/d: adjusted OR = 1.37; *P* < 0.05), elevated fasting glucose (IFG: adjusted OR = 1.46; DM: adjusted OR = 2.32; *P* < 0.001), hypertension (DBP: adjusted OR = 1.58; *P* < 0.001), bigger body size (overweight: adjusted OR = 2.61, obese: adjusted OR = 3.55; *P* < 0.001), or abdominal obesity (large waist: adjusted OR = 1.41, hypso-WHR: adjusted OR = 1.25; *P* < 0.05) were more inclined to have high TG levels than those without these conditions; however, the trend was opposite for women (adjusted OR = 0.41; *P* < 0.001). Performing light physical or mental labour (adjusted ORs = 1.61 and 1.52, respectively; *P* < 0.001), having an elevated fasting glucose (DM: adjusted OR = 1.45; *P* < 0.05), bigger body size (overweight: adjusted OR = 1.98, obese: adjusted OR = 2.95; *P* < 0.001), or abdominal obesity (large waist: adjusted OR = 1.45, hypso-WHR: adjusted OR = 1.21; *P* < 0.05) had an increased risk for low HDL-C; however, having an increased age (adjusted OR = 0.80; *P* < 0.05), female sex (adjusted OR = 0.18; *P* < 0.001), higher monthly family income (4000–6000 yuan: adjusted OR = 0.67; *P* < 0.001), drinking status (adjusted OR = 0.79; *P* < 0.05), or increased alcohol consumption (50 ~ g/d and 75 ~ g/d: adjusted ORs = 0.36 and 0.58, respectively; *P* < 0.05) had the reverse tendency.

**Table 6:** Multivariate stepwise logistic regression analysis of factors associated with dyslipidemia

Variable	Characteristics	High TC (≥5.2 mmol/L)		High LDL-C(≥3.4 mmol/L)		Low HDL-C(<1.0 mmol/L)		High TG(≥1.7 mmol/L)	
		Adjusted OR	*P*	Adjusted OR	*P*	Adjusted OR	*P*	Adjusted OR	*P*
		(95% CI)		(95% CI)		(95% CI)		(95% CI)	
Age (years)	<40	1		1		1		1	
	≥40	1.10(0.94, 1.29)	0.225	1.11(0.94, 1.31)	0.224	0.80(0.69, 0.93)	0.005	0.97(0.84, 1.12)	0.66
Gender	Male	1		1		1		1	
	Female	1.22(0.97, 1.53)	0.084	0.81(0.63, 1.03)	0.08	0.18(0.14, 0.25)	<0.001	0.41(0.32, 0.52)	<0.001
Marital status	Single							1	
	Married							1.43(1.06, 1.93)	0.019
Monthly income	≤4000	1				1			
(RMB, Yuan)									
	4000–6000	1.03(0.87, 1.22)	0.755			0.67(0.56, 0.81)	<0.001		
	>6000	0.76(0.64, 0.91)	0.003			1.07(0.88, 1.30)	0.487		
Working type	Intense physical workers			1		1		1	
	Light physical workers			1.19(0.99, 1.45)	0.071	1.61(1.33, 1.93)	<0.001	1.51(1.28, 1.78)	<0.001
	Mental workers			1.35(1.05, 1.74)	0.02	1.52(1.22, 1.90)	<0.001	1.58(1.30, 1.93)	<0.001
Workplace	Underground front-line			1					
	Underground auxiliary			0.96(0.78, 1.19)	0.737				
	Ground			1.22(0.96, 1.54)	0.101				
	Office			1.16(0.86, 1.56)	0.326				
Length of service	≤3 years	1		1					
	4–10 years	1.23(0.79, 1.93)	0.36	1.38(0.86, 2.19)	0.181				
	>10 years	1.70(1.12, 2.58)	0.013	1.77(1.14, 2.74)	0.011				
Smoking status	No	1							
	Yes	1.25 (1.06, 1.46)	0.007						
Smoking index	0 ~			1					
(roots*years)									
	150 ~			1.39(1.13, 1.71)	0.002				
	300 ~			1.28(1.00, 1.62)	0.048				
	450 ~			1.42(1.16, 1.75)	<0.001				
Drinking Status	No					1		1	
	Yes					0.79(0.67, 0.95)	0.01	1.24(1.05, 1.46)	0.011
Daily drinking(g)	0 ~	1				1		1	
	25 ~	1.53(1.16, 2.00)	0.002			0.80(0.57, 1.11)	0.185	1.01(0.75, 1.35)	0.954
	50 ~	1.81(1.30, 2.53)	<0.001			0.36(0.22, 0.59)	<0.001	0.73(0.51, 1.04)	0.083
	75 ~	1.73(1.30, 2.32)	<0.001			0.58(0.40, 0.84)	0.004	1.37(1.01, 1.87)	0.044
FBG	NFG(<5.6 mmol/L)	1		1		1		1	
	IFG(5.6 mmol/L ≤ FBG < 7.0 mmol/L)	1.41(1.14, 1.75)	0.002	1.20(0.96, 1.50)	0.103	0.89(0.70, 1.13)	0.322	1.46(1.18, 1.81)	<0.001
	DM(≥7.0 mmol/L)	1.99(1.43, 2.78)	<0.001	1.61(1.16, 2.24)	0.005	1.45(1.03, 2.06)	0.036	2.32(1.61, 3.32)	<0.001
DBP	<90 mmHg	1		1				1	
	≥90 mmHg	1.23(1.05, 1.46)	0.013	1.30(1.10, 1.53)	0.002			1.58(1.34, 1.86)	<0.001
BMI	normal weight, 18.5–23.9 kg/m^2^	1		1		1		1	
	underweight, <18.5 kg/m^2^	0.99(0.65, 1.52)	0.962	0.68(0.42, 1.12)	0.129	0.58(0.31, 1.08)	0.088	1.13(0.73, 1.74)	0.594
	overweight, 24.0–27.9 kg/m^2^	1.43(1.22, 1.67)	<0.001	1.77(1.51, 2.08)	<0.001	1.98(1.65, 2.36)	<0.001	2.61(2.23, 3.06)	<0.001
	obesity, ≥28.0 kg/m^2^	1.66(1.37, 2.02)	<0.001	2.35(1.93, 2.86)	<0.001	2.95(2.33, 3.72)	<0.001	3.55(2.85, 4.41)	<0.001
WC	<102 cm (male)/88 cm (female)					1		1	
	≥102 cm (male)/88 cm (female)					1.45(1.14, 1.85)	0.003	1.41(1.12, 1.78)	0.004
WHR	≤0.9(male)/0.8 (female)					1		1	
	>0.9(male)/0.8 (female)					1.21(1.02, 1.42)	0.026	1.25(1.08, 1.45)	0.003

*TG* triglyceride, *TC* total cholesterol, *HDL-C* high-density lipoprotein cholesterol, *LDL-C* low density lipoprotein cholesterol, *FBG* fasting blood glucose

*NFG* normal fasting glucose, *IFG* impaired fasting glucose, *DM* diabetes mellitus, *SBP* systolic blood pressure, *DBP* diastolic blood pressure, *BMI* body mass index, *WC* waist circumference, *WHR* waist/hip ratio

## Discussion

In accordance with a World Health Organization report, of the 56 million deaths worldwide in 2012, 38 million deaths (about 68%) were caused by non-infectious diseases, which have become a severe worldwide public health burden [25, 26]. Generally, CVD is the most outstanding condition globally, and dyslipidaemia is an important public health problem, with an increasing prevalence worldwide. As dyslipidaemia is one of the most crucial independent reversible risk factors for CVDs, preventive measures targeting the popular with dyslipidaemia can reduce the morbidity and mortality of CVDs [27]. To effectively prevent CVDs, lifestyle modifications, policy changes, and pharmacotherapy are required. The current study is one of the largest scale occupational, population-based studies on dyslipidaemia that comprehensively assessed the prevalence of dyslipidaemia and the associated risk factors of dyslipidaemia in coal miners in China.

The average serum TC, LDL-C, and TG levels have increased quickly in China [28–30]. Findings from the 2002 Chinese National Nutrition and Health Survey (CNHS) [28, 31] demonstrated that the respective mean levels of TC, LDL-C, and TG were 3.81 mmol/L, 2.21 mmol/L, and 1.10 mmol/L. The China National Diabetes and Metabolic Disorders Study demonstrated that the respective mean levels of TC, LDL-C, and TG were 4.72 mmol/L, 2.68 mmol/L and 1.57 mmol/L [32]; the corresponding average levels observed in the current study were higher: 5.01 mmol/L, 3.15 mmol/L, and 1.90 mmol/L, respectively. Furthermore, in our study, the average level of serum HDL-C was somewhat lower (1.21 mmol/L) than that (1.30 mmol/L) reported in the two aforementioned studies [28, 31, 32].

Our current study’s findings indicated that 68.28% of Chinese coal miners (men, 71.36%; women, 51.68%) had at least one type of dyslipidaemia. This value is higher than that reported by the 2002 CNHS (18.6%) [28] and by recent studies on Chinese adults [27, 33, 34]. Our findings showed that the prevalence of dyslipidaemia is high in coal miners. In line with the results of previous studies [27, 33, 34], which found that the main types of dyslipidaemia in China are high TG levels, our study showed that the major contributor of dyslipidaemia was high TG levels (40.46%). In the past 20 years, the most prominent change in the concentrations of serum lipids in the people of Beijing and Guangzhou in China was an increase in the TG levels, possibly as a result of the increase in carbohydrates and fats in their diet [35–37], whereas the most common lipid disorder in Western countries was high TC levels [38]. Furthermore, China has a low mortality due to coronary heart disease, and the relationship between a low serum TC level and low fat and cholesterol dietary intake was identified as the main cause of this finding [39]. However, with economic growth and westernized lifestyle, especially dietary patterns, the prevalence of high TC levels increased in China [27, 34], and the prevalence of a high TC level (38.08%) found in this study was significantly higher than the average Chinese national level [33]. Projected trends in the increased TC level would lead to an increase of approximately 9.2 million cardiovascular events from 2010 to 2030 [40]. The association between regional disparities in the type of dyslipidaemia and lifestyle, diet, and other factors requires further investigation. Our findings of the high prevalence of dyslipidaemia in coal miners highlight crucial public health implications what could explain why this population is susceptible to CVDs [15].

In addition to the prevalence of dyslipidaemia, our study identified the potential risk factors for each type of dyslipidaemia: type of work, hypertension, BMI, WC, etc. Previous studies have reported an association between age and dyslipidaemia [27, 29, 38, 41]. The mechanisms underlying the role of age on serum lipid panel results may be hereditary characteristics, degenerative processes, weight gain, and gradual development of insulin resistance [38]. In the current study, univariate logistic regression analysis demonstrated that the prevalence of high TC levels, LDL-C levels, and TG levels significantly increased with advanced age (≥40 years); however, the prevalence of low HDL-C levels decreased, which was in accordance with the results of another previous study [42]. However, multivariate analysis only confirmed the relationship of age and low HDL-C levels. Socio-demographic factors, in line with previous studies, were consistently associated with the prevalence of dyslipidaemia [27, 29, 33]. Multivariate analysis in this study also showed that a married status was an independent risk factor for a high TG level; however, female sex was inversely related with the prevalence of the low HDL-C and high TG levels, and a high monthly income was inversely associated with the prevalence of a high TC and low HDL-C level.

Regarding working conditions, we found that underground front-line workers had the lowest prevalence of each kind of dyslipidaemia, and workers who did intense physical work had the lowest prevalence of dyslipidaemia, because the former undertake work of a higher labour intensity. Results of multivariate logistic regression analysis showed that those who did light physical and mental work were inclined to suffer high LDL-C and TG levels and a low HDL-C level than those who did intense physical work. Furthermore, in univariate logistic regression analysis, compared to underground front-line workers, ground workers or office workers and underground auxiliary workers, who frequently but not continuously work under the coal mine, had higher ORs for the prevalence of all dyslipidaemia components; however, multivariate analysis did not confirm these relationships. Results of multivariable logistic regression analysis in this study showed that the extended length of service was an independent risk factor for high TC and high LDL-C levels. Contrary to the expected results, participants working inside the coal mine did not show a high prevalence of dyslipidaemia, possibly because of the healthy worker effect and the underground front-line workers may be younger, stronger, and in good health.

With regard to health behaviours, smoking is a main health issue in China; the prevalence of smoking was 57.69% in our study. Results of multivariate logistic regression analysis also demonstrated that smoking status was associated with high TC levels. With regard to the smoking index, a positive association was shown between a smoking index and high LDL-C levels. Drinking is also a main health issue in China; the prevalence of drinking was 41.49% in our study. There was an inverse relationship between drinking status and low HDL-C levels in our study, which was in line with the findings of previous research studies [42, 43], and the trend was the opposite for a high TG level. Regarding alcohol consumption per day, an inverse relationship found for low HDL-C levels, although a positive relationship was noted for the prevalence of high TC and high TG levels.

Regarding the health condition, metabolic disturbances, including pathoglycaemia, hypertension, overweight/obesity, abdominal obesity, and dyslipidaemia, occur almost simultaneous with each other and are intensively related to subsequent CVD; the most common underlying mechanism is insulin resistance [44]. Hypertension and diabetes have been reported as risk factors for dyslipidaemia [38, 45–47]. In our study, the prevalence rate of hypertension was as high as 31.38%, and that of IFG/ DM was 14.0%. The increase of dyslipidaemia could have contributed to the high prevalence of hypertension and diabetes among our subjects. Results of multivariate logistic regression analysis showed that participants with elevated fasting glucose levels (IFG/DM) were positively related with all types of dyslipidaemia components; furthermore, a high DBP was a risk factor for high TC levels, high LDL-C levels, and high TG levels. These results proposed that the factors causing dyslipidaemia and the outcomes of dyslipidaemia are similar among people of different ethnicities. Thus, the rising prevalence of dyslipidaemia in China could cause analogous cardio-cerebral-vascular outcomes as it did in Western countries [29].

The findings of this study are in line with several previous studies [38, 45, 46, 48–50] that demonstrated that the prevalence of dyslipidaemia rises with an increase in the BMI; additionally, we found that all types of dyslipidaemia were associated with the accumulation of adipose tissue. The abnormal blood lipid panel in overweight and obese people is probably caused by insulin resistance [44]. In China, 3.8% of men and 5.0% of women were obese in 2013 [51], but this proportion was almost 20% in some cities [52]. The respective prevalence rates of overweight and obesity were 39.00 and 17.55% among coal miners in our study; these values are higher than the Chinese national average values. On the basis of our study’s data, we interpreted that obesity is at a high prevalence among coal miners, and it will undoubtedly affect the prevalence of dyslipidaemia. Therefore, obesity in this professional group needs to be noticed. In our research, we found that the prevalence of abdominal obesity among coal miners in China was 13.50%. Other researchers have observed associations between dyslipidaemia and abdominal obesity [43, 53], and our study found that a large WC and hypso-WHR were risk factors for low HDL-C levels and high TG levels. In summary, dyslipidaemia, particularly high TG is a very common and important health problem among Chinese coal miners. Demographic characteristics (advanced age, male sex, marital status, and monthly family income); working characteristics (working type and length of service); lifestyles (smoking, smoking index, drinking status, and alcohol consumption per day); and health conditions (elevated fasting glucose, hypertension, obesity, and abdominal obesity) were closely related to dyslipidaemia. Effective occupational health education must be provided and urgent occupational protective measures must be taken to control the epidemic of dyslipidaemia among coal mine occupational groups.

Several limitations may exist in our study. First, as this was a cross-sectional study, the results are prone to be affected by the recall bias and unmeasured confounding factors. Second, other risk factors (e.g., environmental air pollutants, dietary intake, and other health conditions) were not considered. Finally, this was a cross-sectional study, so no causal relationships could be precisely delineated.

## Conclusions

Our current study provides dependable and present epidemic characteristics on dyslipidaemia among Chinese coal miners. Dyslipidaemia, especially high TG levels, is a very prevalent and major health issue among Chinese coal miners.

## Abbreviations

BMI: Body mass index
CNHS: Chinese National Nutrition and Health Survey
CVD: Cardiovascular disease
DBP: Diastolic blood pressure
DM: Diabetes mellitus
FBG: Fasting blood glucose
HDL-C: High-density lipoprotein cholesterol
IFG: Impaired fasting glucose
LDL-C: Low-density lipoprotein cholesterol
NFG: Normal fasting glucose
OR: Odds ratio
SBP: Systolic blood pressure
TC: Total cholesterol
TG: Triglyceride
WC: Waist circumference
WHR: Waist-hip ratio
